# Prognostic significance of MyD88 expression by human epithelial ovarian carcinoma cells

**DOI:** 10.1186/1479-5876-10-77

**Published:** 2012-04-25

**Authors:** Yi Zhu, Jian-Ming Huang, Guo-Nan Zhang, Xiao Zha, Bi-Fang Deng

**Affiliations:** 1 Department of Gynecologic Oncology, Sichuan Cancer Hospital, Sichuan, People’s Republic of China; 2 Department of Biochemistry & Molecular Biology, Sichuan Cancer Institute, Sichuan, People’s Republic of China; 3 Graduate School, Guangxi Medical University, Guangxi, People’s Republic of China; 4 Department of Gynaecologic Oncology, Sichuan Cancer Hospital, No. 55, Section 4, South People’s Road, Chengdu, 610041, Sichuan, People’ s Republic of China

**Keywords:** Ovarian cancer, Myeloid differentiation factor 88, Toll-like receptor 4, Prognostic factors, Metastasis

## Abstract

****Background**:**

MyD88 is an adaptor protein for TLR-4 signaling known to mediate paclitaxel resistance in epithelial ovarian carcinoma (EOC). This study examined the clinical significance of MyD88 expression in EOC.

****Methods**:**

MyD88 and TLR-4 expression were examined by immunocytochemistry in 109 specimens of ovarian tissues, comprising EOC (N = 83), borderline tumors (N = 9), benign cysts (N = 9) and normal ovarian tissue (N = 8), and clinical data collected by a retrospective chart review. The correlations between MyD88 expression and clinicopathological factors and outcomes were analyzed.

****Results**:**

TLR-4 expression was detected frequently in all the ovarian tissues. Distinct MyD88 expression was showed in EOC (64 of 83, 77.1 %), in borderline tumors (5 of 9, 55.6 %) and in benign cysts (3 of 9, 33.3 %), and normal ovarian tissue showed no MyD88 expression. Positive MyD88 expression significantly correlated with shorter disease-free and overall survival for EOC (P < 0.0001 and P = 0.0031), and high MyD88 expression was significantly correlated with tumor metastasis (P = 0.0012) for EOC. Univariate and multivariate analyses revealed that MyD88 expression was an independent prognostic factor for disease-free survival and overall survival for EOC.

****Conclusion**:**

Our data indicate that MyD88 expression is a significantly poor prognostic factor for EOC. A better understanding of the role of MyD88 expression in disease progression and outcome may be helpful for development of novel chemotherapies for patients with EOC.

## **Background**

Epithelial ovarian cancer (EOC) is one of the most lethal malignant tumors in women, and the first leading cause of death from gynaecological cancers [1,2]. Some 85 % to 90 % of ovarian cancers are epithelial, and more than two thirds are diagnosed at an advanced stage. Cytoreductive surgery followed by paclitaxel/platinum-based combination chemotherapy is still current management strategies for EOC [3]. Although many tumors initially respond to chemotherapy, patients with metastatic and/or relapsed disease continue to have extremely poor survival outcomes [4,5].

The innate immune system recognizes the presence of bacterial pathogens through the expression of a family known as Toll-like receptors (TLRs) [6].TLRs recognize microbial-associated or host-associated pathogen-associated molecular patterns (PAMP), which leads to the production of proinflammatory cytokines [7,8]. Chronic infection and inflammation are considered to be some of the most important epigenetic and environmental factors contributing to tumorigenesis and tumor progession [9]. Myeloid differentiation protein 88 (MyD88), a TLRs signaling adaptor protein, is an essential downstream component of the TLRs signalling cascade, and recent studies point to a critical role for MyD88 in the protumorigenic inflammatory response [10,11]. It has demonstrated that MyD88 is a novel, cell-autonomous role in RAS signalling, cell-cycle control and cell transformation through its interaction with activated Erk [12].

Recent evidence has identified the contribution of TLR-4MyD88 signaling pathway to epithelial ovarian carcinogenesis, development and a poor response to paclitaxel chemotherapy. Upon TLR-4 ligation with paclitaxel [13], EOC cells that express MyD88 constitutively secrete pro-inflammatory cytokines, including interleukin (IL)-6, IL-8, vascular endothelial growth factor (VEGF) and monocyte chemotactic protein (MCP), which mediates tumor progression, invasion, metastasis and paclitaxel chemoresponse [14-17]. However, little is known about the clinicopathological factors and prognostic significance of MyD88 expression in EOC. Investigation of MyD88 status is needed to differenciate those patients with EOC. By detecting levels of MyD88 expression, it might be possible to identify those patients with EOC most likely to benefit from adjuvant chemotherapy and target treatment accordingly. Therefore, a better understanding of MyD88 as a potential marker is needed to further optimize treatment strategies, to develop new chemotherapeutic agents and to evaluate prognostic significance of EOC.

The objective of this study was to investigate the prognostic significance of immunohistochemical detection of MyD88 expression and its association with clinicopathological factors in patients with EOC.

## **Methods**

### **Tumer cell lines**

Human EOC cell lines SKOV-3 and A2780 were purchased from the Committee on Type Culture Collection of Chinese Academy of Sciences (CTCCCAS,Shanghai, China). Cell lines were maintained in culture in 75 cm flasks with RPMI 1640 medium (GIBCO, Invitrogen), supplemented with 2 mmol/L L-glutamine, 10 % heat-inactivated fetal calf serum (FCS), 100 U/ml penicillin and 40U/ml Gentamicin, in a humidified atmosphere of 5 % CO2 and 95 % air. Subconfluent cells (80 %) were passaged with a solution containing 0.25 % trypsin and 0.5 mmol/L EDTA.

### **Patients and human tissues**

A total of 109 patients who underwent surgery from 1999 to 2009 at the Sichuan Cancer Hospital were investigated in this study. Information on patient demographics (age) and tumor features (pathology, histological grade, FIGO stage, lymph node metastasis, malignant cells in ascites, liver or lung metastasis, and residual tumor) was obtained from clinical and pathological records (Table 1). Disease stages were classified according to the criteria proposed by the FIGO (International Federation of Gynecology and Obstetrics). Tissue collection was done before chemotherapy. All patients received six to eight cycles of paclitaxel/carboplatin (TP) regimen after surgery. Exclusion criteria were cancers associated with sex cord-stromal tumors, germ cell tumor, or secondary tumors. All patients, enrolled in this study, had signed informed consent, approved by our Internal Ethical Committee.

**Table 1:** Clinicopathological features according to TLR4 and MyD88 expression

**Clinicopathological factors**		**TLR4**			**MyD88**			**MyD88+**	
	**High**	**Low**		**Positive**	**Negative**		**High**	**Low**	
	**(n = 67 )**	**(n = 16 )**	**P-value**	**(n = 64 )**	**(n = 19 )**	**P-value**	**(n = 28 )**	**(n = 36 )**	**P-value**
Age(years)									
≥55	32	11	0.1311	29	14	0.0268	11	18	0.2743
<55	35	5		35	5		17	18	
Pathology									
Serous	50	12	0.6249	46	15	0.3850	21	25	0.4190
Other	17	4		18	4		7	11	
Histological grade									
Well /Moderate	23	6	0.5135	25	4	0.1192	15	10	0.0328
Poor/ Clear cell	44	10		39	15		13	26	
FIGO Stage									
I, II	11	1	0.2742	9	3	0.5528	5	4	0.3393
III, IV	56	15		55	16		23	32	
Malignant cells in ascites									
Yes	34	4	0.0555	32	6	0.1240	12	20	0.2250
No	33	12		32	13		16	16	
Lymph node metastasis									
Yes	20	2	0.1345	20	2	0.0610	15	5	0.0008
No	47	14		44	17		13	31	
Liver or lung metastasis									
Yes	25	4	0.2666	27	2	0.0087	16	11	0.0298
No	42	12		37	17		12	25	
Residual tumor									
>1	32	6	0.3244	32	6	0.1240	14	18	0.5993
<1	35	10		32	13		14	18	

Abbreviations: TLR4 = Toll-like receptor 4; MyD88 = myeloid differentiation factor 88. Bold values indicate P < 0.05.

For the immunohistochemical study, formalin-fixed, paraffin-embedded tissue samples from normal ovarian tissue (n = 8) obtained by oophorectomy, as well as benign cysts (n = 9), borderline tumors (n = 9), and EOC (n = 83), were used.

### **SDS-PAGE and Western blots**

SKOV-3 and A2780 cells were lysed in RIPA buffer [1 % Triton X-100, 150 mmol/L NaCl, 1 mmol/L EGTA, 50 mmol/L Tris–HCl, 0.1 % sodium dodecyl sulfate (SDS), 1 % sodium desoxycholate and phenylmethylsuphonyl fluoride (PMSF)] and disrupted by sonication on ice. Cell lysates were centrifuged at 12 000 rpm for 20 min at 4°C, the supernatant was used for TLR4 and MyD88 protein determination by western blots. Twenty μg of total protein from each cell lysate ( Bradford assay ) were separated on a 8 % ( for TLR4) and 12 % ( for MyD88) polyacrylamide/SDS gel. After protein transfer (BIO-RAD Trans-Blot® SD for 30 min) to a polyvinylidene difluoride membrane (BIO-RAD Sequi-BlotTM PVDF membrane, US), the membranes were incubated in blocking buffer for 1 hour and then incubated with rabbit anti-human TLR4 and MyD88 Abs (dilution 1:1000; Epitomics and Abcam, US) overnight at 4°C. After washing, the membranes were incubated with a horseradish peroxidase-labeled goat antirabbit IgG (1:10 000 dilution; SANTA, US) for 1 hour at room temperature. For protein detection, membranes were incubated with ECL-substrate for 5 min (CoWin Biotech, Beijing, China). The band intensities were digitalized and quantified using the ChemiDoc XRS + software (Bio-Rad, US).

### **Immunohistochemistry for TLR4 and MyD88**

SKOV-3 and A2780 cells were grown on covered glass slides in culture medium for 48 hours. Cells were fixed with methyl alcohol and acetone (1:1) for 10 min at −20°C; Paraffin section of tumor tissues (4-μm) were deparaffinized in xylene and rehydrated in a series of ethanols, and antigen retrieval was performed using an autoclave oven technique. The slides were then placed in a staining dish with 0.3 percent H_2_O_2_ to quench for endogenous peroxidase activity for 30 min and washed with PBS three times. The area around the tissue sections or cell slides was scored with a Pap pen to limit the amount of antibodies and reagents used. All steps occurred at room temperature with the slides placed in a moisture chamber to keep the tissue or cell from drying out during the procedure. To block for non-specific background, 100–200 μl of 5 % BSA made in PBS were added to the circumscribed areas and incubated for 20 min in the moisture chamber. Next, the primary antibody (4 – 5 μg/ml) was incubated overnight at 4°C with the primary antibody (4 – 5 μg/ml) followed by washing with PBS three times, the specimens were incubated with biotinylated anti-rabbit IgG (5 μg/ml) and horseradish peroxidase streptavidin (4 μg/ml) for 50 min at 37°C. The color was developed with DAB (2.5 mg 3, 3’-diaminobenzidine in 5 ml 0.1 mol/L Tris). Twenty five μl of 0.03 percent H_2_O_2_ were added to the chromogen just before use. The slides were washed with double distilled water and counterstained lightly with hematoxylin and mounted in glycerol jelly. Human SKOV-3 cell line was used as a positive control for TLR4 and MyD88 immunoreactivity. PBS without the primary antibody served as negative control.

### **Evaluation of immunohistochemical findings**

Each slide was evaluated independently by two pathologists who were blinded to clinical and outcome data. As most samples stained for TLR4 and MyD88 showed similar color intensity, from moderate to strong, no evaluation of color intensity was performed in this study. Any intensity of membrane and/or cytoplasmic staining was considered to represent a positive stain for TLR4 and MyD88. Several high-power fields (×400) selected from different staining density regions including high, moderate, low, and negative staining areas were captured using a digital camera (Olympus IX71 Inverted Fluorescence Microscope and AnalySIS image capture software). Photographs were printed on plain paper and a grid was drawn over them. We counted a mean of 2 000 tumor cells per tumor (range, 1 500 – 2 500), and results were expressed as the percentage of tumor cells with a positive stain. Thereafter, the percentage of TLR4- or MyD88-positive tumor cells was scored on a scale of 0 – 4 (0: no staining; 1+: ≤10 %; 2+: 11-30 %; 3+: 31-50 %; 4+: >50 %). Furthermore, the expression levels of TLR4 and MyD88 were divided into the follow two groups according to score: low (1+, 2+) and high (3+, 4+).

### **Statistical analysis**

Analysis was performed using SPSS 17.0 software. The Pearson χ2 exact test or Fisher’s exact test was used to compare qualitative variables. The primary statistical outcomes were DFS and OS measured from the day of surgery. DFS was defined as the interval between the day that surgery was performed and the day that recurrence was first detected. If recurrence was not diagnosed, the date of death or of last follow-up was used. OS was defined as the interval between the dates of surgery and death. The follow-up period after the initial operation for the primary lesion was 5 years for DFS and OS. Both DFS and OS were estimated by Kaplan-Meier curves, and the curves were compared using the log-rank test. Time to relapse and to death was analyzed using the Cox proportional hazards model for univariate and multivariate analyses. In addition, the hazard ratios (HRs) between prognostic groups and their 95 % confidence intervals were computed. Probability values (P) < 0.05 were considered to be statistically significant.

## **Results**

### **Expression of TLR4 and MyD88 in ovarian cancer cell lines and tissues**

**Figure 1:**
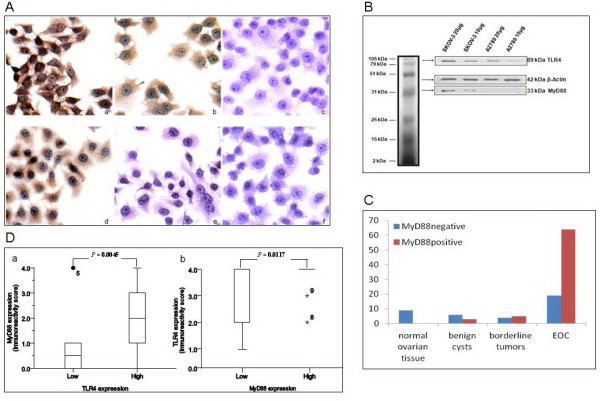
** A. TLR4 and MyD88 expression in EOC cell lines.** Immunostaining for TLR4 protein in SKOV-3 (a) and A2780 (d) (×400). Immunostaining for MyD88 protein in SKOV-3 (b) and A2780 (e) (×400). Negative stainging for TLR4 and MyD88 using PBS (c, f). B. Expression of TLR4 and MyD88 in SKOV-3 and A2780 cell lines by using Western blot assays. C. The expression rates of MyD88 were gradually increased with the progress ovarian lesions. The positive rates of MyD88 in normal ovarian tissue, benign cysts, borderline tumors and EOC were 0, 33.3 %, 55.6 % and 77.1 %. D. (a) High expression of TLR4 was correlated with high expression of MyD88 in EOC. EOC with a high expression of TLR4 (N = 67) showed significantly high levels of MyD88 expression than did EOC with a low expression of TLR4 (N = 16) (P = 0.0045). (b) EOC with a high expression of MyD88 (N = 28) showed significantly high levels of TLR4 expression than did EOC with a low expression of MyD88 (N = 55) (P = 0.0117).

The TLR4 and MyD88 expression were evaluated in human EOC cell lines and tumor sections. Positive immunoreactivity for TLR4 was observed in SKOV-3 and A2780 and positive immunoreactivity for MyD88 only in SKOV-3 by staining of cell crawled-slides (Figure 1A) and by western blotting (Figure 1B). No staining was observed when PBS was used as a negative control. The SKOV-3 cell line was obtained from the ascites of a patient with advanced, metastatic EOC. SKOV-3 is resistant to most cytotoxic drugs [18]. In contrast, the A2780 cell line was derived from a primary untreated and paclitaxel-sensitive cancer [19].

**Figure 2:**
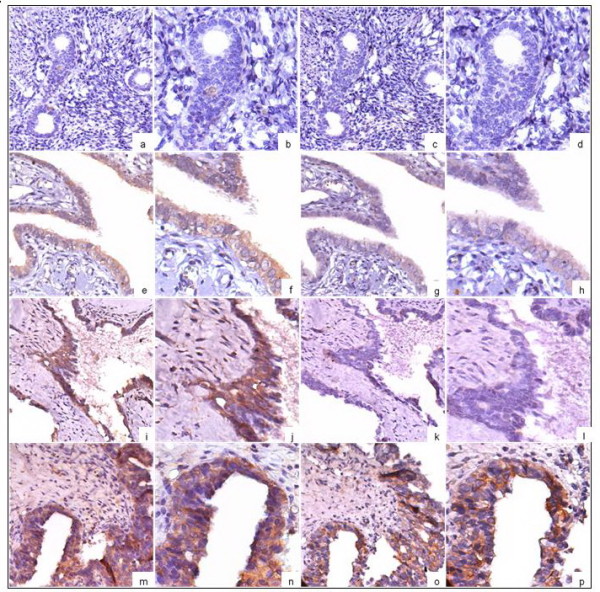
** Expression of TLR4 and MyD88 in normal ovarian tissue, benign cysts, borderline tumors and EOC.** Detection of TLR4 and MyD88 immunoreactivity in normal ovarian tissue (a-d), benign cysts (e-h), borderline tumors (i-l), and EOC (m-p). Original magnification, (a, c, e, g, i, k, m, o) × 200; (b, d, f, h, j, l, n, p) × 400. Normal ovarian tissue showed no or weak immunoreactions for TLR4 (a, b). Strong immunostaining of TLR4, which was localized in the membrane or cytoplasm, was observed in the benign cysts (e, f), borderline tumors (i, j) and EOC (m, n). In contrast to the situation in the normal ovarian tissue (c, d), benign cysts (g, h), borderline tumors (k, l), strong immunostaining of MyD88, which was localized in the cytoplasm, was vserved in EOC (o, p).

Immunolocalization of TLR4 protein was observed in the membrane and cytoplasm. In general, TLR4 was found to be expressed in normal ovarian epithelium (Figure 2a, b), benign cysts (Figure 2e, f), borderline tumors (Figure 2m, n) and malignant tumors (Figure 2i, j). However, the intensity of staining for TLR4 in malignant tumors was considerably greater than that in normal tissues or benign tumors. Normal ovarian epithelium did not express TLR4 signaling adapter protein, MyD88 (Figure 2c, d). MyD88 expression was observed in benign cysts, but greatest in borderline and malignant tumours (Figure 1C). In EOC, a total of 64 of 83 (77.11 %) cancers showed MyD88 expression, but a variably strong or weak signal (4+: 9, 3+: 19, 2+: 18, 1+: 18, 0: 19) (Figure 2o, p).

In the co-expression analysis, MyD88 expression levels were higher in TLR4 high-expression EOC than in TLR4 low-expression EOC (P = 0.0045, Figure 1D-a). Similarly, significantly high levels of TLR4 were detected in EOC with high MyD88 expression (P = 0.0117, Figure 1D-b). Finally, the correlation between the expression of TLR4 and MyD88 in EOC was confirmed using Pearson’s correlation coefficient analysis (r = 0.28, P = 0.0093) and Spearman’s correlation coefficient analysis (P = 0.0093). In addition, 40 (48 %) cancers showed a high-level combined expression of TLR4/MyD88 (6+: 17, 7+: 15, 8+: 8).

### **Clinicopathological significance of TLR4 and MyD88**

The co-distribution of EOC with a differentiated TLR4/MyD88 expression in relation to cancer and patient characteristics is shown in Table 1 and Table 2. The high expression of MyD88 was significantly associated with histological grade (P = 0.0113), lymph node metastasis (P = 0.0001) and liver or lung metastasis (P = 0.0029). Furthermore, expression of MyD88 was significantly associated with age (P = 0.0268).

**Table 2:** Clinicopathological features according to differentiated MyD88 expression

	**Negative**	**Low**		**Negative**	**High**		**Negative + low**	**High**	
**Clinicopathological factors**	**(n = 19 )**	**(n = 36 )**	**P-value**	**(n = 19 )**	**(n = 28 )**	**P-value**	**(n = 55 )**	**(n = 28 )**	**P-value**
Age(years)									
≥55	14	18	0.0787	14	11	0.0208	32	11	0.0811
<55	5	18		5	17		23	17	
Histological grade									
Well /Moderate	4	10	0.4202	4	15	0.0256	14	15	0.0113
Poor/ Clear cell	15	26		15	13		41	13	
Lymph node metastasis									
Yes	2	5	0.5412	2	15	0.0026	7	15	0.0001
No	17	31		17	13		48	13	
Liver or lung metastasis									
Yes	2	11	0.0888	2	16	0.0012	13	16	0.0029
No	17	25		17	12		42	12	

Abbreviations: MyD88 = myeloid differentiation factor 88. Bold values indicate P < 0.05.

### **Clinicopathological parameters and patient survival in EOC**

At the 5-years follow-up, 49 patients had recurrence (DFS rate: 59.0 %), and 35 patients had died (OS rate: 42.2 %). In univariate analysis, FIGO stage (DFS, P = 0.0137; OS, P = 0.0368), malignant cells in ascites (DFS, P = 0.0242; OS, P = 0.1913), lymph node metastasis (DFS, P = 0.0001; OS, P = 0.0076), liver or lung metastasis (DFS, P < 0.0001; OS, P = 0.0003) and residual tumor (DFS, P = 0.0164; OS, P = 0.0950) were important factors associated with DFS and OS. Patient age, pathology and histological grade were not related to DFS or OS (Table 3A). Multivariate analysis was performed to identify independent prognostic factors. Models that include all histopathological variables and tumor markers found to have significant prognostic value in univariate analysis are shown. FIGO stage was significantly associated with DFS (P = 0.0446) and OS (P = 0.0468), and the presence of liver or lung metastasis was significantly associated with DFS (P = 0.0380) and OS (P = 0.0237) (Table 3B).

**Table 3:** Clinicopathological features, tumor markers, and patient survival (univariate analysis)

**Variable**	**5-Year DFS HR (95 % CI)**	**P-value**	**5-Year OS HR (95 % CI)**	**P-value**
**A. univariate analysis**				
Age (≥55 years vs <55 years)	0.77(0.43-1.36)	0.3659	0.76(0.38-1.51)	0.4313
Pathology (serous vs other)	0.51(0.24-1.09)	0.0833	0.69(0.29-1.67)	0.4100
Histological grade (poor vs well/moderate)	0.61(0.34-1.07)	0.0859	0.73(0.37-1.43)	0.3587
FIGO stage (III/IV vs I/II)	5.95(1.44-24.55)	0.0137	8.41(1.14-62.03)	0.0368
Malignant cells in ascites (yes vs no)	1.96(1.09-3.51)	0.0242	1.59(0.79-3.20)	0.1913
Lymph node metastasis (yes vs no)	3.29(1.99-6.03)	0.0001	2.55(1.28-5.07)	0.0076
Liver or lung metastasis (yes vs no)	4.28(2.35-7.80)	<0.001	3.62(1.81-7.25)	0.0003
Residual tumor (>1 vs <1)	2.04(1.14-3.66)	0.0164	1.81(0.90-3.63)	0.0950
TLR4 (high vs low)	1.78(0.79-4.02)	0.1657	1.68(0.67-4.19)	0.2685
MyD88 (positive vs negative)	0.10(0.02-0.41)	0.0014	0.09(0.01-0.67)	0.0185
**B. multivariate analysis**				
FIGO stage (III/IV vs I/II)	4.57(1.04-20.17)	0.0446	7.89(1.03-58.90)	0.0468
Malignant cells in ascites (yes vs no)	1.38(0.73-2.60)	0.3255	——	——
Lymph node metastasis (yes vs no)	1.25(0.62-2.52)	0.5315	0.94(0.43-2.05)	0.8713
Liver or lung metastasis (yes vs no)	2.21(1.05-4.69)	0.0380	2.47(1.13-5.41)	0.0237
Residual tumor (>1 vs <1)	1.04(0.54-1.99)	0.9082	——	——
MyD88 (high vs low)	0.11(0.23-0.47)	0.0027	0.12(0.02-0.89)	0.0382

Abbreviations: DFS = disease-free survival; HR = hazard ratio; CI = confidence interval; OS = overall survival; FIGO = International Federation of Gynecology and Obstetrics; TLR4 = Toll-like receptor 4; MyD88 = myeloid differentiation factor 88. Bold values indicate P < 0.05.

### **TLR4 and MyD88 expression and patient survival in EOC**

**Figure 3:**
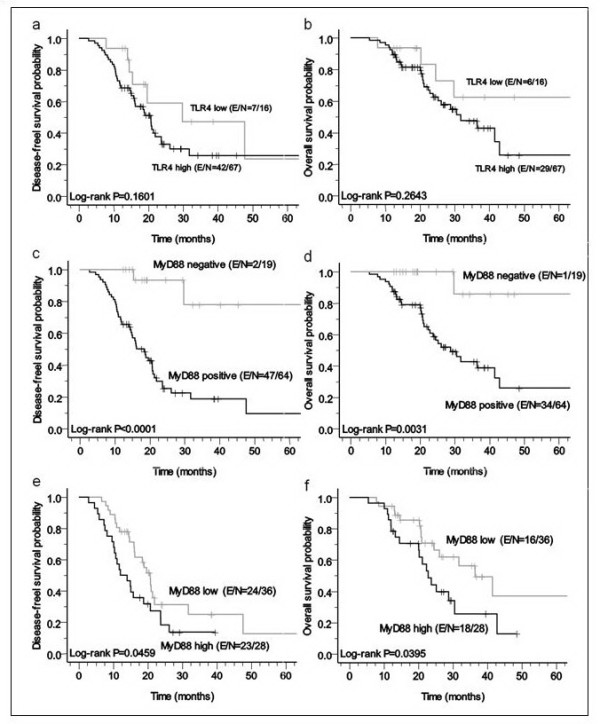
** Kaplan-Meier survival curves of DFS and OS in patients with EOC according to TLR4 and MyD88 expression.** (A, B) High expression of TLR4 was potentially associated with poor DFS and OS (P = 0.1076, P = 0.2158, respectively). (C, D) High expression of MyD88 was significantly associated with poor DFS and OS (P = 0.0007, P = 0.0008, respectively). (E, F) High co-expression of TLR4 + MyD88 was significantly associated with poor DFS and OS (P < 0.0001, P = 0.0057, respectively).

We assessed the influence of expression of TLR4 and MyD88 in EOC on patient survival. Patients with a high TLR4 expression had a worse DFS and OS than did those with a low TLR4 expression (median DFS: 20.73 vs. 29.73 months; median OS: 31.77 vs. 66.30). However, a high expression of TLR4 did not significantly influence DFS (log-rank P = 0.1657; Figure 3a) and OS (log-rank P = 0.2685; Figure 3b). Expression of MyD88 was significantly related to poor DFS (log-rank P = 0.0014, Figure 3c) and OS (log-rank P = 0.0185, Figure 3d). In addition, patients whose tumors expressed low-MyD88 had a statistically significant improved DFS and OS compared with patients whose tumors expressed high-MyD88 (median DFS: 12.02 vs 20.73 months, P = 0.0459; median OS: 22.87 vs. 36.47 months, P = 0.0395, respectively, Figure 3e, f).

In univariate analysis, high expression of TLR4 was not significantly related to DFS and OS (Table 3A). Expression of MyD88 was significantly related to poor DFS (HR: 0.10; 95 % CI: 0.02 – 0.41; P = 0.0014) and significantly influenced OS (HR: 0.09; 95 % CI: 0.01 – 0.67; P = 0.0185). In multivariate analysis, expression of MyD88 was significantly associated with poor DFS (adjusted HR: 0.11; 95 % CI: 0.23 – 0.47; P = 0.0027) and OS (adjusted HR: 0.12; 95 % CI: 0.02 – 0.89; P = 0.0382) (Table 3B).

## **Discussion**

It was recently reported that TLR4/MyD88 signalling drives tumour growth in EOC with MyD88 positive expression. It has been known that TLR4/MyD88 signalling in tumour cells itself has important roles as oncogenic factors [20]. Silencing TLR4/MyD88 signalling in tumour cells results in reduced tumour formation, and the inhibition of tumour-cell apoptosis by MyD88-dependent TLR4 signalling was observed in EOC [19,21]. Recent reports revealed that TLR4/MyD88 signalling drives tumour growth and chemoresistance to paclitaxel in EOC. In addition, MyD88 has been shown to be pivotal for the resistance to paclitaxel and the promotion of tumour progression in patients with EOC [20,22]. TLR4/MyD88 signalling have been shown to be associated with local chronic inflammation [22,23]. Activation of TLR4/MyD88 signalling seems to promote the development of EOC by mechanisms including enhanced expression of proinflammatory cytokines. In this study, we observed that TLR4 expression is ubiquitous in all detected ovarian tissues, and that MyD88 was overexpressed in EOCs compared with borderline tumors and benign cysts, but not expressed in normal ovarian tissues. A subgroup of EOC cells differentially expressing MyD88 has demonstrated enhanced cytokine/chemokine production and cellular proliferation upon activation of TLR4 [14-17].

The molecular pathway that links inflammation to the acquisition of metastatic capacity during tumour progression has been investigated [24]. Recent reports suggest that MyD88 expression of EOC cells may be associated with the invasion and metastasis of tumor by acquisition of epithelial mesenchymal transition(EMT) phenotype. Our study showed that MyD88 overexpression was frequently detected in EOCs with lymph node metastasis and liver or lung metastasis. Our findings suggest that MyD88 overexpression promotes EOC progression by contributing to metastasis. MyD88 positive EOC cells have a functioning TLR4/MyD88 pathway and are possibly indicative of an ovarian cancer stem cell that is highly resistant to pro-apoptotic signalling [25]. Recent reports have identified CD44 as a potential marker for the identification of cancer stem cells in ovarian cancer, and confirmed the functionality of the TLR4-MyD88 pathway in only the CD44^+^ cell population [25,26]. CD44^+^ EOC cells express TLR-4 and MyD88 and respond to TLR-4 ligands by activating NF-κB, promoting a pro-inflammatory microenvironment, and proliferating with chemotherapy,suggesting that the TLR-4/MyD88 pathway may play a critical role in the process of repair/differentiation triggered by the cancer stem cells. Furthermore, ex vivo manipulation of ovarian CSC differentiation can significantly decrease MyD88 expression [27].

Blocking TLR4/MyD88 signalling would provide a survival benefit. However, the association between TLR4/MyD88 signalling and cancer mortality has not been well investigated in clinical samples. Our results clearly demonstrated that overexpression of MyD88 was an independent and significant prognostic factor for DFS and OS. In addition, patients whose tumors expressed MyD88 were younger compared with patients whose did not express MyD88. Recent clinical studies have indicated that low-grade serous EOC is relatively chemoresistant in the primary, neoadjuvant and recurrent settings, which was in agreement with our finding that high expression of MyD88 closely depended upon this tumor type [28]. It has been documented that MyD88 expression is independent of histologic grade and associated with significantly shorter patient survival [27]. These results suggest that MyD88 expression may be more important than histologic subtype or grade in epithelial ovarian cancers but remains to be determined.

MyD88 negative EOC cells lack this functioning pathway that are more responsive to apoptosis and therefore less biologically aggressive [29,30], When MyD88 negative EOC cells are transfected with MyD88, making them positive cells, resistance to paclitaxel is induced. Previous studies reported that elevation of the downstream signals of the TLR4/MyD88 pathway, such as IL-6 and NF-κB was related to EOC patient survival [31]. We see that TLR4/MyD88 regulates the expression of IL-6, which shows its important role in many aspects of tumour growth. NF-κB is an end point of the TLR4/MyD88 signalling pathway. Numerous lines of evidence that link NF-kB activation to cancer development have been reported. Our previous studies showed that an antagonizing TLR4 sesquiterpenoid compound from Atractylodes macrocephala Koidz could downregulate the expression and release of IL-6 induced by paclitaxel or LPS in MyD88 positive SKOV-3 cells, which are more resistant to paclitaxel than MyD88 negative A2780 cells. These findings suggested that the tumour cell TLR4/MyD88 signalling pathway has a crucial role in EOC patient prognosis and that blocking this pathway could provide great benefits for patients with EOC. We evaluated the correlation between MyD88 expression and clinical outcome, and we have found that all patients who had MyD88 positive tumors presented with poor DFS and OS, while the patients with MyD88 negative tumors had an excellent prognostic outcome. Our study profiled the status of MyD88 in ovarian tumour samples, and showed the clinicopathological significance of tumour cell self-MyD88 expression in EOC.

## **Conclusions**

In conclusion, MyD88 overexpression was associated with carcinogenesis and tumor progression of EOC and with an increased risk of metastasis and worse survival, and the identification of MyD88 expression as determined by immunohistochemistry is a useful prognostic factor in EOC. However, Our clinical conclusions about MyD88 expression as a prognostic marker in EOC were based on retrospective analyses and are needed to be independently validated.

## **Competing interests**

The authors declare that they have no competing interests.

## **Authors' contributions**

YZ carried out cell culture and western-blot, immunohistochemistry experiments, participated in data analysis and interpretation, statistical analysis, and wrote the manuscript preparation. JMH participated in experiments, and performed study design and manuscript editing. GNZ mentioned the study concepts and planned the studies and revised the manuscript. XZ participated in quality control of data and algorithms. BFD carried out cell culture and worked on data acquisition. All authors have read and approved the final manuscript.
